# Correlation between gut microbiota characteristics and non-small cell lung cancer based on macrogenomics sequencing

**DOI:** 10.1186/s41065-024-00328-w

**Published:** 2024-08-27

**Authors:** GuiLin Zeng, LiRong Zeng, Ying Wang, Zhi Cao, XiangHua Zeng, ZhiHong Xue, ShiLan Liu, YaMao Li, Lang He

**Affiliations:** 1 Department of Oncology, The Second Clinical Medical College, Affiliated Fifth People’s Hospital of Chengdu, Cancer Prevention and Treatment Institute of Chengdu, Chengdu Fifth People’s Hospital, University of Traditional Chinese Medicine), No.33, Mashi Street, Wenjiang District, Chengdu City, Sichuan Province 611130 China; 2 Chengdu Railway Health School, Chengdu City, Sichuan Province 611741 China

**Keywords:** Cancer, NSCLC, Gut microbiota, Radiotherapy, Macrogenomics sequencing

## Abstract

**Objective:**

Non-small cell lung cancer (NSCLC) patients undergoing chemotherapy and immunotherapy experience disturbances in the gut microbiota. This study intends to find out the correlation between gut microbiota and clinical indices before and after radiotherapy for NSCLC.

**Methods:**

Ten patients with primary NSCLC were screened, and plasma and fecal samples were collected before and after radiotherapy, respectively. Inflammatory indices in plasma were detected. Genomic DNA was extracted from fecal specimens and sequenced on on Illumina HiSeq2000 sequencing platform. Thee sequenced data were subjected to Metagenome assembly, gene prediction, species annotation, and gene function analysis to study and analyze gut microbiota and metabolic functions. The correlation between the diversity of gut microbiota and the clinical indicators of NSCLC patients was evaluated, and the changes of gut microbiota before and after radiotherapy were observed.

**Results:**

The diversity of gut microbiota in NSCLC patients did not correlate with smoking, pathology, and inflammatory markers. The abundance of phylum (p)_*Bacteroidetes* increased; p_*Firmicutes* and p_*Bacteroidetes* accounted for the highest proportion in NSCLC patients, and the abundance of both was dominantly exchanged after radiotherapy. There was a decrease in genus (g)_*Bifidobacterium* after radiotherapy in NSCLC patients. There was no significant correlation between the diversity of gut microbiota after radiotherapy and radiotherapy sensitivity, and the structural composition and abundance of gut microbiota remained stable.

**Conclusion:**

The diversity of gut microbiota is altered after radiotherapy in NSCLC patients, showing an increase in harmful bacteria and a decrease in beneficial bacteria.

**Supplementary Information:**

The online version contains supplementary material available at 10.1186/s41065-024-00328-w.

## Introduction

Nearly 85% of all lung cancer cases are non-small cell lung cancer (NSCLC), which together account for the leading cause of cancer deaths in the world [1]. According to global cancer statistics published by the International Agency for Research on Cancer, there will be an estimated 2.2 million new lung cancer cases and 1.8 million lung cancer-related deaths worldwide in 2020, with approximately 820,000 new lung cancer cases and 715,000 lung cancer-related deaths in China [2, 3]. Most patients miss the optimal treatment time for lung cancer due to its insidious onset, resulting in poor treatment efficacy and a low five-year survival rate of 4–17% [4]. The current method of early lung cancer screening relies mainly on thin-layer chest CT for lung occupation detection, and pathological diagnosis requires further clarification. Early-stage tumors, however, are usually small and metabolically inactive, making bronchoscopy, CT, and PET-CT difficult. The inability to specify the type of pathology delay treatment despite lung occupation being detected in many patients [5, 6]. Therefore, less invasive, economically feasible, easy-to-obtain and more positive screening modalities are needed [7].

The gut microbiota is the most complex and symbiotic microbial ecosystem in the human body [8, 9]. Gut microbiota is a general term for the variety of bacteria that colonize the human gastrointestinal tract. Gut microbiota is widely distributed, complex microbial communities that maintain normal physiological and immune functions of the host’s intestinal tract and catabolize and metabolize food components to make them more readily absorbed. Gut microbiota has a systemic impact on the physiology and health of the host, and alterations in composition, function, or gut microbiota-host interactions are directly associated with diseases [10]. The relationship between lung disease and gut microbiota interactions has been termed the “gut-lung axis” [11, 12], and the gut microbiota is associated with respiratory-related diseases such as acute lung injury [13], exacerbation of chronic obstructive pulmonary disease [14], asthma [15, 16], among other respiratory-related diseases [17]. Through the bloodstream, changes in gut microbiota can affect lung immunity, and vice versa [18]. Gut microbiota dysbiosis can cause a variety of lung diseases; inflammatory lung diseases can cause changes in the structure of gut microbiota; and disturbances in the gut microbiota can affect lung immunity and even promote tumor formation [19]. There is increasing evidence of a significant association between gut microbiota and lung cancer [20, 21]. Studies have reported that the relative abundance of *Prevotella* in lung cancer patients is reduced, and that the abundance of *Actinomycetes* and *Streptococcus* is significantly higher compared with that of healthy populations [22], and significant alterations are found in the intestinal, lung, and upper respiratory tract microbiota after chemotherapy for NSCLC [23]. The gut microbiota associated with checkpoint inhibitor response in human and mouse models of NSCLC is also found to induce changes in the tumor microenvironment [24].

Radiotherapy is one of the main treatment modalities for lung cancer at present, and 77% of lung cancer patients have evidence-based indications for radiotherapy. Along with the improvement of radiotherapy technology and the emergence of anticancer drugs and detection technology, the status and application mode of radiotherapy have been greatly changed in the treatment of NSCLC. Postoperative sequential radiotherapy or synchronized radiotherapy will benefit patients significantly, and elderly patients who cannot tolerate synchronized chemotherapy or refuse chemotherapy due to their mindset can also benefit from simple radiotherapy for lung cancer [25, 26]. After irradiation of the oral cavity and laryngopharynx, the toxic side effects of radiotherapy are closely related to changes in the gut microflora [27]. Fecal bacteria transplantation has a defensive effect on the intestinal damaging properties caused by radiotherapy. *Bifidobacterium*, *Lactobacillus*, and *Streptococcus* have attenuated toxicity such as radiation enteritis and intestinal necrosis after radiotherapy [28]. Radiation-induced oral mucositis in head and neck cancer patients are able to reduce the toxic side effects using oral *Lactobacillus subtilis* CD2 [29].

In this study, we collected fecal samples before and after thoracic radiotherapy from primary NSCLC patients, compared the composition and abundance of gut microbiota by macro-genomics sequencing, and analyzed the correlation between gut microbiota and patients’ clinical information, as well as the gut microbiota changes.

## Materials and methods

### Study subjects

Ten primary patients admitted to Chengdu Fifth People’s Hospital in 2018–2019 who were diagnosed with NSCLC by pathology were selected.

Inclusion criteria: diagnosed with lung cancer by pathological examination; patients not having received radiotherapy, chemotherapy, targeted therapy, or lung cancer surgery; receiving chest radiotherapy; Karnofsky Performance Scale (KPS) score ≥ 80; expected survival > 3 months; greater than 18 years old and less than 80 years old; untreated with antibiotics, corticosteroids, or immunosuppressant drugs for at least the past two months; signed written consent prior to participation in the study.

Exclusion criteria: cognitive difficulties; refusal or inability to give explicit consent to participate; history of gastrointestinal surgery (total or partial gastrointestinal resection, cholecystectomy, etc.); previous infectious diseases requiring antibiotic treatment within 2 months (pneumonia, gastrointestinal tract inflammation, cholecystitis, pancreatitis) within 2 months; oral antibiotics, enteral nutrition, oral probiotics, prebiotics, laxative in the last 21 days.

The study was approved by the Ethics Committee of Chengdu Fifth People’s Hospital, with strict screening criteria, and all patients signed an informed consent form after being informed of the sampling process and detailed study plan. All included patients received a questionnaire survey, which included: permanent place of residence, current residence, gender, age, smoking, alcohol consumption, and drug history. The sample collection was done by the same doctor in Chengdu Fifth People’s Hospital.

## Detection of inflammatory indices

Fasting venous blood of 5–10 mL was drawn from all patients at 6:30 − 7:30 a.m and immediately sent to Chengdu Fifth People’s Hospital for testing. Leukocytes, neutrophils, lymphocytes, monocytes, and platelets were detected by Myriad BC-6800 whole blood cell analyzer, and then the neutrophil/lymphocyte ratio (NLR), lymphocyte/monocyte ratio (LMR), and platelets/lymphocyte ratio (PLR) were calculated.

### Sample collection

Fecal samples were collected before and after 30 Gy of radiotherapy (2 Gy per day, 5 times per week for 3 weeks) and were registered as RB.GUT [1–10] for pre-radiotherapy and RA.GUT [1–10] for post-radiotherapy. The inner part of the middle section of the stool (colonic mucosals exist in the surface layer of the stool) was taken out by the stool interceptor. The fecal samples (2–3 g) were loaded into 3 collection tubes, with each tube containing 1 g. The collected samples were stored at -80℃.

### DNA extraction from fecal samples

Whole genome DNA was extracted from fecal samples using QAmap DNA Mini Kit51306 (Qiagen, Germany). Each 4 µg of DNA was incubated with 160 µL of MBD-Fc-bound glass beads. To reduce host DNA contamination, the host DNA was separated to allow for enrichment of microbial DNA, and the microbial DNA was purified using the ethanol precipitation method. The purified product was measured for concentration, and DNA purity was determined by spectrophotometry A260/A280, agarose gel electrophoresis was used to analyze the fragment size and integrity of the DNA, and the extracted DNA was stored at -80 °C.

### Library construction and high-throughput sequencing

The qualified DNA samples were randomly broken by ultrasonic disrupter and divided into fragments of about 350 bp length. After qualification, different libraries were combined according to the effective concentration and the target data volume, and then sequenced by Illumina Hiseq 2000.

### Data processing

Readfq was used to preprocess the raw data to obtain clean data for subsequent analysis. Low-quality bases were removed, and in cases where host genes cause contamination, the reads from host genes must be filtered out.

### Bioinformatics analysis

Composition analysis: Sequences were aligned with the reference genome (National Center for Biological Information [NCBI]) using SOAP align 2.21 software to determine species composition.

Abundance calculation: Sequences were calculated on the database alignment, the abundance of sequences obtained from the alignment and the abundance of non-specific sequences were combined to obtain the abundance.

Species diversity analysis: Alpha diversity index was used to analyze the diversity of microbial communities, and the diversity of a single sample can be detected in terms of diversity and abundance. Chao 1 index focuses on the relative abundance of microbial communities, while Shannon and Simpson mainly assess the diversity of bacterial microbiota.

### Metagenome assembly

The clean data obtained after data sequencing were then assembled. The assembled Scaffolds were broken from N junctions to obtain N-free sequence fragments called Scaftigs. Fragments below 500 bp are filtered out, and the obtained data were subjected to statistical analysis and gene prediction.

### Gene prediction

ORF prediction was performed based on the assembled Scaftigs, thereafter a non-redundant initial gene catalogue was obtained. The abundance of each gene in the gene catalogue was calculated for each sample, and basic information statistics, core gene analysis and pan gene analysis, correlation analysis, and Venn analysis were performed. Based on the abundance information of the genes in each sample, the overall gene number of each sample was obtained. A variable number of samples were randomly selected to obtain the number of genes between samples, and dilution curves were constructed and plotted for the Core and Pan genes to compare the differences in gene composition between groups and to judge the rationality of the sample selection. A box plot of gene number differences between groups was plotted. Venn Graph was used to examine the distribution of the number of identical and different genes between groups, and to analyze the common and unique information of genes between different samples.

### Species annotation

The obtained Unigenes were compared to species from NCBI’s NR database for the following species: bacteria, fungi, archaea, and viruses. Species were analyzed in Kingdom, Phylum, Class, Order, Family, Genus, and Species levels to display relative abundance. Anosim inter- (intra-) analysis, Metastat analysis of inter-group species, and LEfSe multivariate statistical analysis were conducted.

### Annotation of commonly used functional databases

Unigenes was compared with each functional database, and the comparison yielded data from abundance tables at each level to perform Metastat and LEfSe analyses of functional differences between groups based on functional abundance, and comparative analyses of metabolic pathways.

### Statistical methods

SPSS 21.0 statistical software was used to analyze the data, which were expressed as mean ± standard deviation. Normality of the measures was tested using the Shapiro-Wilk test, and comparisons of different subgroups whose measures satisfied normality were performed using the independent samples t test or one-way ANOVA. Comparisons between two groups that did not satisfy normality were made using the independent sample Mann-Whitney U test and the Wilcoxon signed rank test. The chi-square test was used for comparison of count data and Spearman Rank correlation test was used for correlation analysis. Comparison of diversity indices was tested by Student’s t test. Differences were considered statistically significant at *P* < 0.05.

## Results

### Basic clinical conditions of patients

A total of 10 patients were included in this experiment (Table 1) including 6 males and 4 females; 4 squamous carcinomas and 5 adenocarcinomas; most of the patients were staged as IIIA-IIIC (8 patients). There were 5 patients who were below the median and 5 patients who were above the median of NLR, PLR and LMR, respectively.

**Table 1:** Included baseline demographic and clinical characteristics of patients

Indicators		No. of patients	%
Gender	Male	6	60%
	Female	4	40%
Age (years)	> 66	5	50%
	< 66	5	50%
Smoking	Yes	6	60%
	No	4	40%
Pathology	Squamous carcinoma	4	40%
	Adenocarcinoma	5	50%
	Others	1	10%
Stage	IIIA-IIIC	8	80%
	IVA	2	20%
Prognosis	PR	6	60%
	PD	4	40%
PFS	> 90	6	60%
	< 90	4	40%
NLR	> 2.78	5	50%
	< 2.78	5	50%
PLR	> 152	5	50%
	< 152	5	50%
LMR	> 2.78	5	50%
	< 2.78	5	50%

*Note* TNM staging was performed according to the AJCC 8th edition of lung cancer standard. Progression free survival (PFS) is defined as the time from diagnosis to disease progression or follow-up. Follow-up: Patients were followed up after the end of treatment, and patients were followed up regularly through outpatient or telephone. The follow-up deadline was October 2019

### Correlation of gut microbiota diversity with clinical indicators in patients

**Fig. 1:**
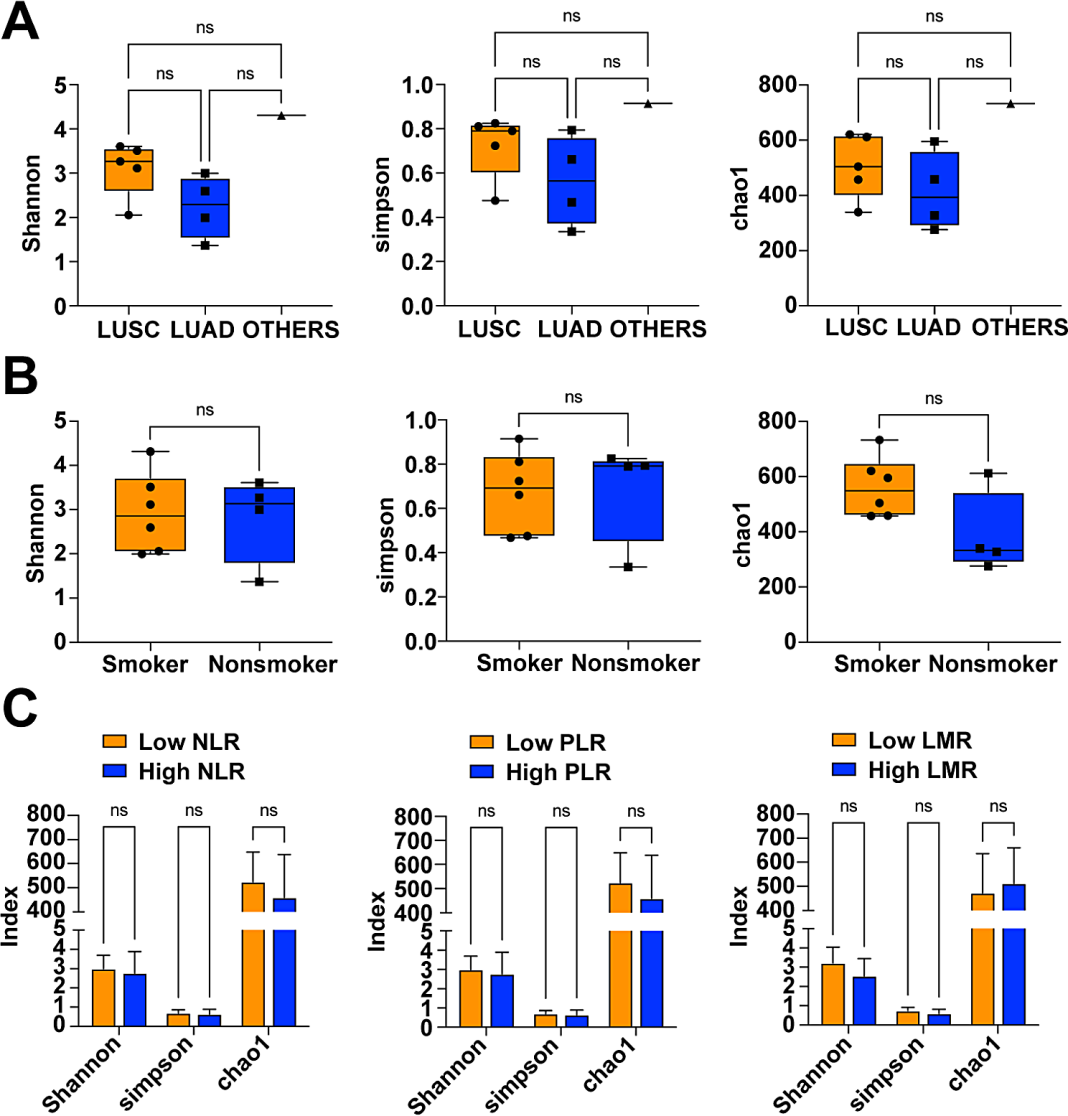
Correlation analysis between the diversity of gut microbiota and patients’ clinical indicators. **A**, Comparison of Shannon, Simpson, and chao1 indices in patients with different pathological types; **B**, Comparison of Shannon, Simpson, chao1 indices in patients with different smoking status; **C**, Comparison of Shannon, Simpson, chao1 indices in patients with different NLR, PLR, and LMR levels. ns *P* > 0.05, * *P* < 0.05

Alpha diversity includes the number of species contained in the community, that is, species abundance; the relative density of each species in the community indicates species evenness. After analyzing the sequencing data from the 10 samples before treatment and further comparing the differences in species diversity, the Shannon and Simpson indices and the chao1 index were introduced, and the results showed that there was greater variability in the diversity indices between the samples (Supplementary Table 1). The difference in Shannon, Simpson, and chao1 indices was not statistically significant across pathology types (Fig. 1A), and the same was true across smoking situations (Fig. 1B). Thus the diversity of gut microbiota was not associated with pathological type and smoking history in NSCLC patients. The patients were divided into low NLR, PLR and LMR group and high NLR, PLR and LMR group according to the median of NLR, PLR and LMR, respectively, and the analysis found that the difference of Shannon, Simpson, chao1 indices was not statistically significant in different NLR, PLR or LMR groupings (Fig. 1C), and that the diversity of gut microbiota was not related with the inflammatory indicators in NSCLC patients.

### Genetic analysis of gut microbiota before and after radiotherapy

**Fig. 2:**
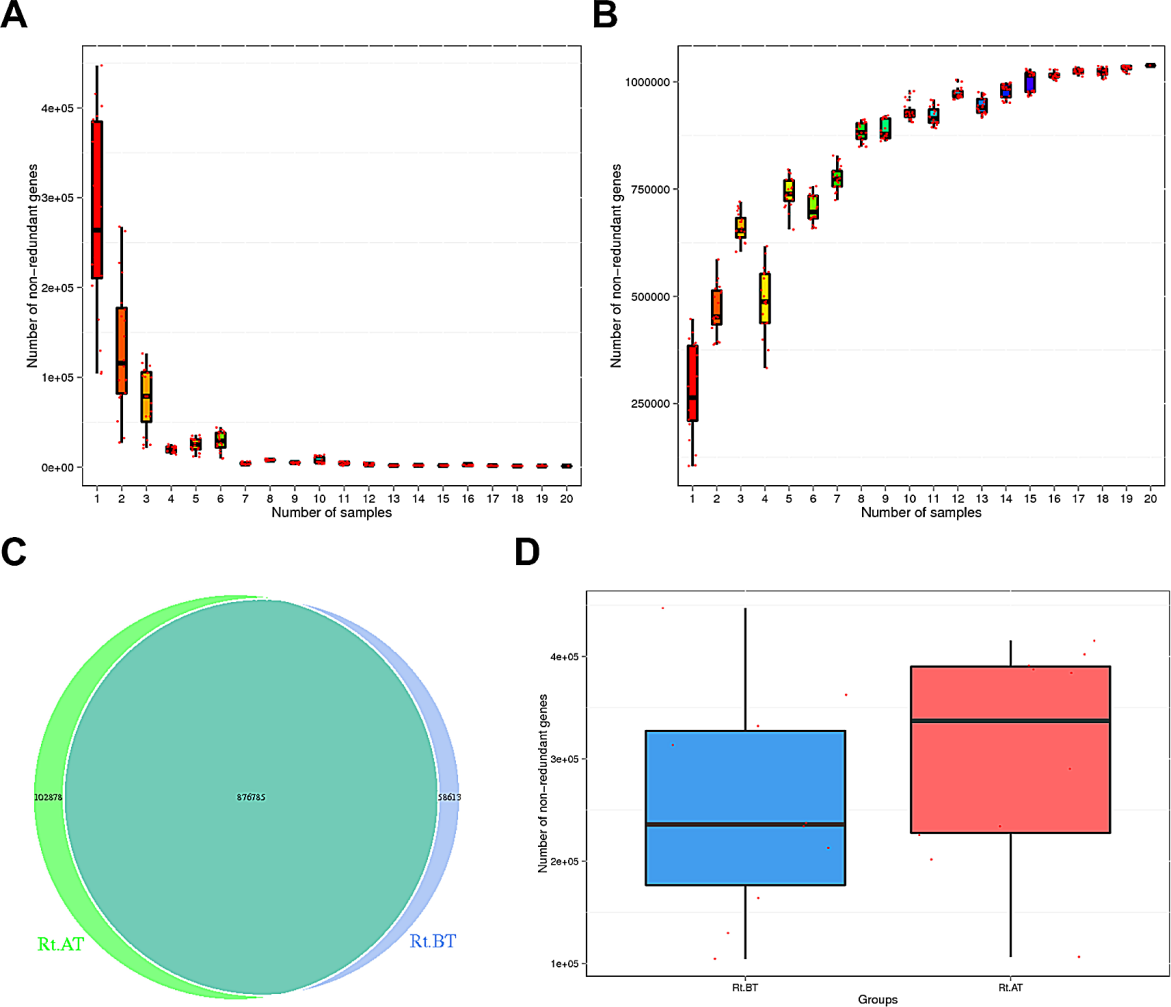
Genetic analysis of gut microbiota before and after radiotherapy. **A**, Core gene curve; **B**, Pan gene curve; **C**, Intergroup gene Venn plots; **D**, Intergroup gene box plots. **A**-**B**, horizontal coordinates indicate the number of samples taken; vertical coordinates indicate the number of genes in the combination of samples taken

The correlation between gut microbiota and NSCLC radiotherapy was studied. The fecal samples of NSCLC patients were divided into pre-radiotherapy (Rt.BT group) and post-radiotherapy (Rt.AT group). First, the differences in gene composition between groups were compared at the genetic level and the rationality of sample selection was judged. Core and Pan gene curves were produced. The core gene is the intersection of genes common to all samples, and the number of genes detected in a given random sample from 20 stool samples from NSCLC patients was decreasing as the number of samples increased; the curve showed a downward trend (Fig. 2A). The pan gene is the concatenation of genes contained in all the samples. As seen from the pan gene curve (Fig. 2B), 20 samples were screened. With the increase of the number of randomly selected samples, the number of genes gradually increased, showing a smooth curve. Then the curve tended to be straight, which would not change with the increase of the number of samples. It was found that the total number of genes in the pre- and post-radiotherapy groups was 876,786, genes unique to the Rt.BT group (pre-radiotherapy) were 586,613, and genes unique to the Rt.AT group (post-radiotherapy) were 1028,878 (Fig. 2C). The same genes accounted for a larger number of genes, the link between the groups was greater, and there was some similarity between the two groups. The fundamental reason being that the fecal sample was collected from the same patient at different times. However, there was some variability in the number of genes between the two groups; both median, high, and low values in the post-radiotherapy groups increased compared to pre-radiotherapy group (Fig. 2D).

### Species analysis of gut microbiota before and after radiotherapy

There were significant changes in the number of species levels before and after radiotherapy. The number of species level of the 4th and 8th patients decreased slightly, while increased in other 8 patients, especially at the species level, but all the differences were not statistically significant (Supplementary Fig. 1, Supplementary Table 2). Although up-regulation of the Shannon, Simpson and chao1 indices could be seen, the differences between the groups were not statistically significant (Supplementary Fig. 2, Supplementary Table 1). In addition, the results of comparing the differences in Shannon, Simpson, and chao1 indices across treatments showed no statistical significance (Supplementary Fig. 3).

**Fig. 3:**
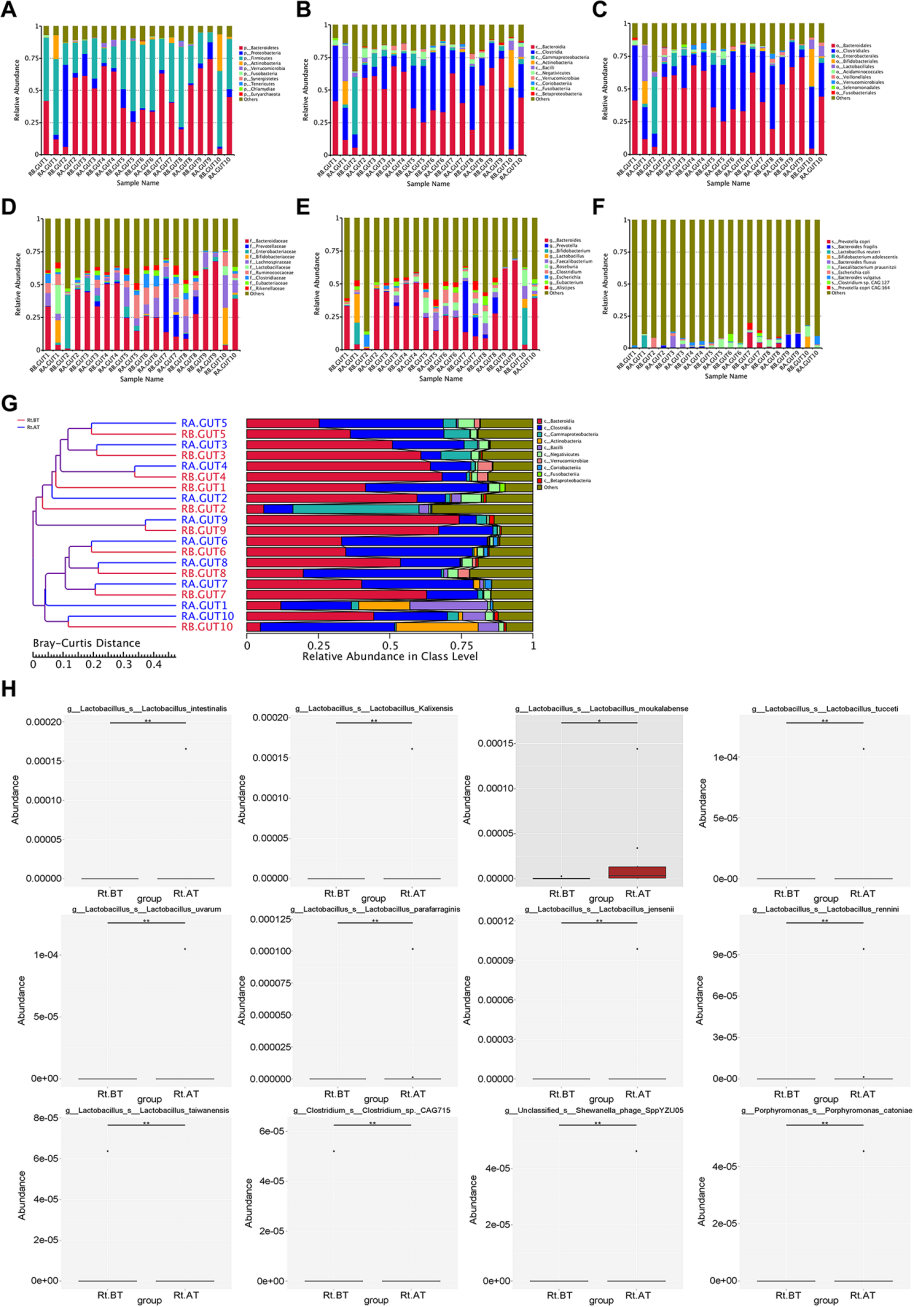
Species analysis of gut microbiota before and after radiotherapy. **A**, Relative abundance of species at the phylum level; **B**, Relative abundance of species at the class level; **C**, Relative abundance of species at the order level; **D**, Relative abundance of species at the family level; **E**, Relative abundance of species at the genus level; **F**, Relative abundance of species at the species level; **G**, Cluster analysis graph at the phylum level based on the abundance of the species; H, Box plots displaying the significantly differing species. * *P* < 0.05, and ** *P* < 0.01

According to the relative abundance table of species, the top 10 species in each sample were listed, and the species that could not be compared with the gene database were set as Others. The relative abundance histogram was drawn to show the abundance of species annotation results corresponding to each sample at different taxonomic levels (Fig. 3A-F). At the same time, the clustering tree of the samples was constructed. The Bray-Curtis distance is one of the most commonly used index in the systematic clustering method, and it is mainly used to portray the degree of proximity between the samples. Clustering diagrams of the species structure and abundance of each sample at the phylum, class, and order levels showed that after the same treatment, the bacterial structure of the same specimen was highly consistent with that before treatment (Fig. 3G). In order to compare species with significant differences between groups, we compared species abundance data using the Metastats method and plotted box plots. Significant species differences are shown in Fig. 3H. There were seven species that differed significantly at the phylum level: five phyla that appeared after radiotherapy (*Candidatus Buchananbacteria*,* Rhodothermaeota*,* Candidatus Levybacteria*,* Candidatus Nealsonbacteria*), and two phyla that decreased or disappeared in abundance after radiotherapy (*Thermodesulfobacteria*,* Candidatus Pacebacteria*). There were 23 strains that differed at the family level: 16 families that appeared or increased after radiotherapy and 6 families that decreased or disappeared in abundance after radiotherapy. At the family level, the increase in abundance of *Lactobacillaceae* was particularly pronounced, with a preradiotherapy percentage of 1.2% and a postradiotherapy increase of 2.5% (1.213% vs. 2.524%, *p* = 0.067). There were 26 strains that differed at the genus level: 19 genera that appeared or increased after radiotherapy and 12 genera that decreased or disappeared in abundance after radiotherapy. Post-radiotherapy abundance of each sample revealed a decrease in abundance after radiotherapy in the genera *Prevotella* and *Bifidobacterium*, but none of them were statistically different. There were 25 species that differed at the species level: 19 species that appeared or increased after radiotherapy and 6 species that decreased or disappeared in abundance after radiotherapy. There was a decrease in abundance of *Prevotella* species and an increase in abundance of *Lactobacillus Reuteri* species after radiotherapy, which was not statistically significant but the trend was still a decrease in *Prevotella* species and an increase in *Lactobacillus* species.

### Differential gene KEGG signaling pathway enrichment analysis

**Fig. 4:**
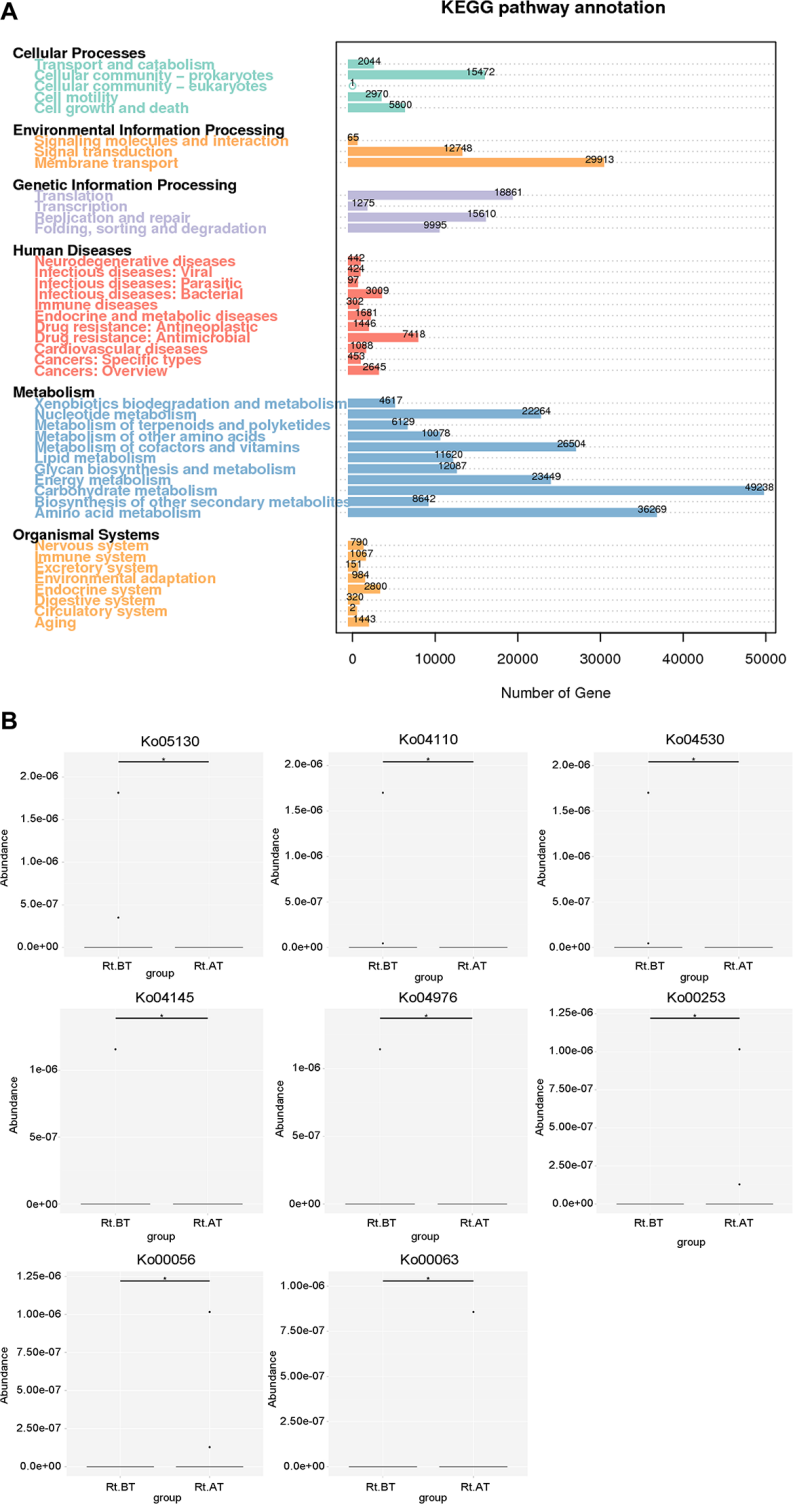
Enrichment analysis of KEGG signaling pathway of differential genes. **A**, Statistical graph of the number of KEGG-annotated genes; **B**, Box plot display of differential species. * *P* < 0.05, and ** *P* < 0.01

The sample genes were compared with KEGG database to get the metabolic pathway annotation information. The results of the analysis are shown in Fig. 4A: lung cancer gut microbiota genes involved many functional pathways, among which the genes for carbohydrate metabolism and amino acid metabolism were the most numerous functional genes of gut microbiota, which accounted for 49,238 and 36,269, respectively. In addition, membrane transport, metabolism of cofactors and vitamins, energy metabolism, nucleotide metabolism, translation, and replication were the most abundant functional genes in gut microbiota, accounting for 29,913, 26,504, 23,449, 22,264, 18,961, and 15,610, respectively. After statistical analysis, the number of increased KOs in the intestinal microecology of the pre-radiotherapy and post-radiotherapy groups were 86 and 125, respectively, and the number of decreased KOs were 27 and 50. By annotating the differentially expressed KOs to KEGG module, it was found that the functional genes enriched in the post-radiotherapy group were distributed in the phosphoenolpyruvate-dependent sugarphosphotransferase system (PTS system) and nucleotide sugar biosynthesis. Annotation of the differentially expressed KOs to the KEGG secondary classification level showed no significant difference between the two groups. At the tertiary level, as shown in Fig. 4B, differences were found between groups in tetracycline biosynthesis and type II polyketide backbone biosynthesis pathways in individual cases.

## Discussion

*I*ncreasing evidence suggested that gut microbiota is involved in the efficacy and toxicity of tumor therapy [30]. In this study, we used macrogenomics to detect gut microbiota in fecal samples before and after radiotherapy in 10 patients with NSCLC. The results of our preliminary analysis of the 10 patients can be reached at the level of the species, with the lowest number of species at 1103 and a maximum of 2957. The species level of this experiment was less than 10%, and about 90% of microorganisms still lack reference genes to analyze. At the base of the species that could not be annotated, the proportion of each level was as follows: at the level of phylum, 10%, 15% at the level of order, 20% at the level of order, 36% at the level of family, and about 50% at the level of genus. Many of these species that could not be annotated were not known bacteria, which lacked the gene and genome information to compare and contrast with each other, and the role of their functions needed to be further explored.

Microbial diversity index is one of the important indicators of gut microbiota balance. NLR, LMR, and PLR are commonly used in the clinical diagnosis of NSCLC as auxiliary indicators of inflammatory response [31, 32]. It was found that there was no significant correlation between alpha diversity and NLR, PLR, and LMR. Moreover, it was also found that there was no significant correlation between alpha diversity and other clinical characteristics of patients. To analyze the causes contributing to the aforementioned outcomes. It was considered that the enrolled patients were fewer in number, and the enrolled cases were all lung cancer patients, and the intra-group differences were greater than the inter-group differences, which could not reflect the changes in alpha diversity in the case of unifactorial grouping. Moreover, due to the lack of studies on the effects of dynamic changes in inflammatory factors and the production of gut microbiota, the mechanism of interaction is lacking.

We compared the sequencing data with the MicroNR database to obtain species annotation information for genes (Unigene) and combined it with a gene abundance table to annotate the 20 specimens studied (pre- and post-radiotherapy) at six levels: phylum, class, order, family, genus, and species. Among the top 10 species ranked by gut microbiota, 98% of them could be attributed to the following four: *Bacteroides*, *Actinomycetes*, *Mycetozoan*, and *Firmicutes*. However, the comparative analysis before and after treatment showed that the abundance of gut microbiota changed considerably, and our results demonstrated that radiotherapy did cause gut microecological dysregulation. Gut microbiota are found to be greatly altered after environmental changes, with diet being the most significant [33, 34]. After 24 h of feeding, the abundance and structure of the gut microbiota will change [35]. After the human body ingests different nutrients, the gut microbiota will interact with each other and maintain homeostasis without destroying its overall structure and function [36–38]. In this experiment, the interval between the first fecal sample sampling and the second sampling was 20–30 days. All patients were hospitalized during radiotherapy. The eating place was mostly selected as the hospital canteen. The diet structure changed greatly compared with that before hospitalization, and the psychological factors were not the same. However, from the Bray-Curtis distance matrix for inter-sample cluster analysis plots, it can be seen that the relative abundance of species at the phylum, genus, and species levels was relatively similar for specimens taken at different times from each patient in the dynamic study. Na et al. [39] selected nine gynecologic oncology patients who underwent pelvic radiotherapy, and compared the gut microbiota of the patients at different time periods during the radiotherapy process with that of the six healthy controls, and found that the gut microbiota of cancer patients before radiotherapy was identical to that of the controls in terms of several species with the highest abundance at the phylum level, but the abundance of each phyla appeared to be significantly different compared with that of the controls. Similar conclusions were reached in this experiment, in which specimens from the same patient before and after treatment had the highest similarity at different levels. This suggests that the intestinal microecology can still maintain a certain degree of stability after experiencing different external factors.

Further analysis of the differences in gut microbiota diversity in patients before and after radiotherapy revealed that although the diversity indices increased after radiotherapy, they were not statistically significant. ANOSIM analysis showed that the intra-group differences were greater than the inter-group differences. It may be due to the same source of specimens before and after treatment, and the difference in the proportion of gut microbiota between different individuals is more obvious. Gender, age, weight, BMI, psychological factors, and different dietary habits can lead to different gut microbiota structures. Because different individuals are in different social environments, the gut microbiota are quite different. This suggests that changes in gut microecology may be more influenced by area of residence, race, or age relative to differences in microecology before and after lung cancer treatment. Nevertheless, the microbial composition of the post-radiotherapy group showed some variability compared to the pre-radiotherapy group. The diversity and stability of the gut microbiota of infants gradually increases, and basically reaches the adult level at the age of 2–3 years, with *Bacteroidetes* and *Firmicutes* as the dominant phyla, and *Fusobacteria*,* Actinobacteria*,* Spirochaetes*,* Proteobacteria*,* Tenericutes*,* Verrucomicrobia*, and *Cyanobacteria* as the predominant phyla [40]. The present study showed that *Bacteroides* (40.56%), *Firmicutes* (34.6%), *Proteobacteria* (10.9%), and *Actinobacteria* (3.3%) were dominant. It has been shown that the proportion of *Bacteroidetes* and *Firmicutes* in the gut microbiota of obese individuals shows a significant decrease [41]. A mutually reinforcing symbiotic relationship exists between both *Bacteroides* and *Firmicutes*, which can work together to promote the absorption or storage of energy in the host. *Bacteroides* and *Firmicutes* in the digestive tract are important for the fermentation of polysaccharides, and even more important is the ratio of the two; a decrease in *Bacteroides* or an increase in *Firmicutes*, and a decrease in the ratio of *Bacteroides* to *Firmicutes*, can contribute to obesity in the host. In our study, the ratio of *Bacteroides/Firmicutes* was reversed after radiotherapy, but there was no significant change in body weight or dynamic change in BMI in patients, and this change in ratio before and after treatment needs to be emphasized and dynamically monitored for clinical changes. Metastat showed that the main dominant species remained stable.

The abundance of gut microbiota changed after radiotherapy. The trend of change in species abundance after radiotherapy was that all levels of species showed an increase in *Lactobacillus* and a significant decrease in *Prevotella* and *Bifidobacterium* after radiotherapy. A recent study has also showed that the abundance of *Lactobacillus* increased significantly after chemotherapy compared with that before chemotherapy in NSCLC [42]. *Lactobacillus* is generally considered beneficial and could serve as a potential radioprotective agent in intestinal injury induced by radiotherapy [43]. Alteration of *Lactobacillus* plays a critical role in radiotherapy of NSCLC. But the exact implications of these changes require further investigation since it was also reported reduced after radiotherapy [44]. *Bifidobacterium* is known for its role in preventing intestinal inflammation, enhancing barrier function, and producing vitamins [45]. Reduced abundance of *Bifidobacterium* after radiotherapy may affect these beneficial roles and has important health implications [46]. *Prevotella* can recognize and synthesize lipopolysaccharide through Toll⁃like receptors (TLR) 4, activate the MyD88/NF-κB signaling pathway, thereby promotes the production of inflammatory response and secretes immune cytokines [47]. Hence complicating the interpretation of its reduced abundance. Radiation causes healthy tissue toxicity as a side effect and the small intestinal epithelium is a major site of injury during radiation therapy. Resulting in radiation enteritis and gut microbiota dysbiosis [48]. Reduced abundance of *Prevotella* and *Bifidobacterium* after radiotherapy could reflect ectopic gut microbiota and disruption of the gut microbiota structure, which characterized by a significant reduction in the abundance of anaerobic bacteria (such as *Prevotella* and *Bifidobacterium*) [46]. Overall, the relationship between the gut microbiome and radiotherapy of NSCLC is bidirectional, multifactorial, and complex. Gut microbiota alterations caused by radiotherapy may be an outcome, but may also be a factor affecting the outcome of radiotherapy.

The meaningful species found from all levels of the species were compared with the patients with PR and PD in the evaluation of tumor efficacy, and no rules were found. Whether it is related to the prognosis of patients needs further study. Due to the small number of cases in this study, the data did not show significant statistical significance, but some valuable information can still be obtained from the trend of data changes, and more case samples need to be collected for verification.

## Conclusion

In summary, our study found that the diversity of gut microbiota in NSCLC patients would be changed after chest radiotherapy, and the trend of change was an increase in harmful bacteria and a decrease in beneficial bacteria. However, the correlation between the differences in gut microbiota before and after radiotherapy and the efficacy of radiotherapy as well as the prognosis of patients needs to be analyzed and studied by collecting more pathological samples.

### Electronic supplementary material

Below is the link to the electronic supplementary material.

Supplementary Material 1

## Data Availability

Data is available from the corresponding author on request.
