# Supplementary material for: Correlation between gut microbiota characteristics and non-small cell lung cancer based on macrogenomics sequencing
Source: Hereditas. 2024 Aug 27;161:26. doi: 10.1186/s41065-024-00328-w (PMC11348753; doi:10.1186/s41065-024-00328-w)
Supplement: Supplementary file 1 — Supplementary Material 1 [file 41065_2024_328_MOESM1_ESM.docx]

Supplementary Table 1 The diversity index of each sample

| Sample | Shannon | Simpson | chao1 |
| --- | --- | --- | --- |
| RB.GUT1 | 3.114037158 | 0.723174069 | 457.5 |
| RA.GUT1 | 3.749711768 | 0.846817665 | 598 |
| RB.GUT2 | 3.607643117 | 0.825560892 | 339.3333333 |
| RA.GUT2 | 2.427425727 | 0.536043969 | 472.5 |
| RB.GUT3 | 2.059189321 | 0.475409578 | 504.3636364 |
| RA.GUT3 | 3.168482856 | 0.728710217 | 522.5 |
| RB.GUT4 | 1.994562164 | 0.467011816 | 458.6470588 |
| RA.GUT4 | 2.045685969 | 0.489451954 | 435.1176471 |
| RB.GUT5 | 3.268710933 | 0.789989531 | 612.1 |
| RA.GUT5 | 3.954368432 | 0.866391269 | 649.5833333 |
| RB.GUT6 | 3.512006421 | 0.810896168 | 621 |
| RA.GUT6 | 3.645976657 | 0.83248276 | 624.2727273 |
| RB.GUT7 | 2.59392564 | 0.661805699 | 595.3703704 |
| RA.GUT7 | 4.062804404 | 0.895701411 | 651.7727273 |
| RB.GUT8 | 4.309216792 | 0.914850823 | 732.75 |
| RA.GUT8 | 3.480833572 | 0.798694844 | 692.5555556 |
| RB.GUT9 | 1.371379798 | 0.3356341 | 276 |
| RA.GUT9 | 0.816396624 | 0.175885957 | 295.9285714 |
| RB.GUT10 | 2.995966436 | 0.794248997 | 327.5454545 |
| RA.GUT10 | 2.902252508 | 0.671945209 | 387 |


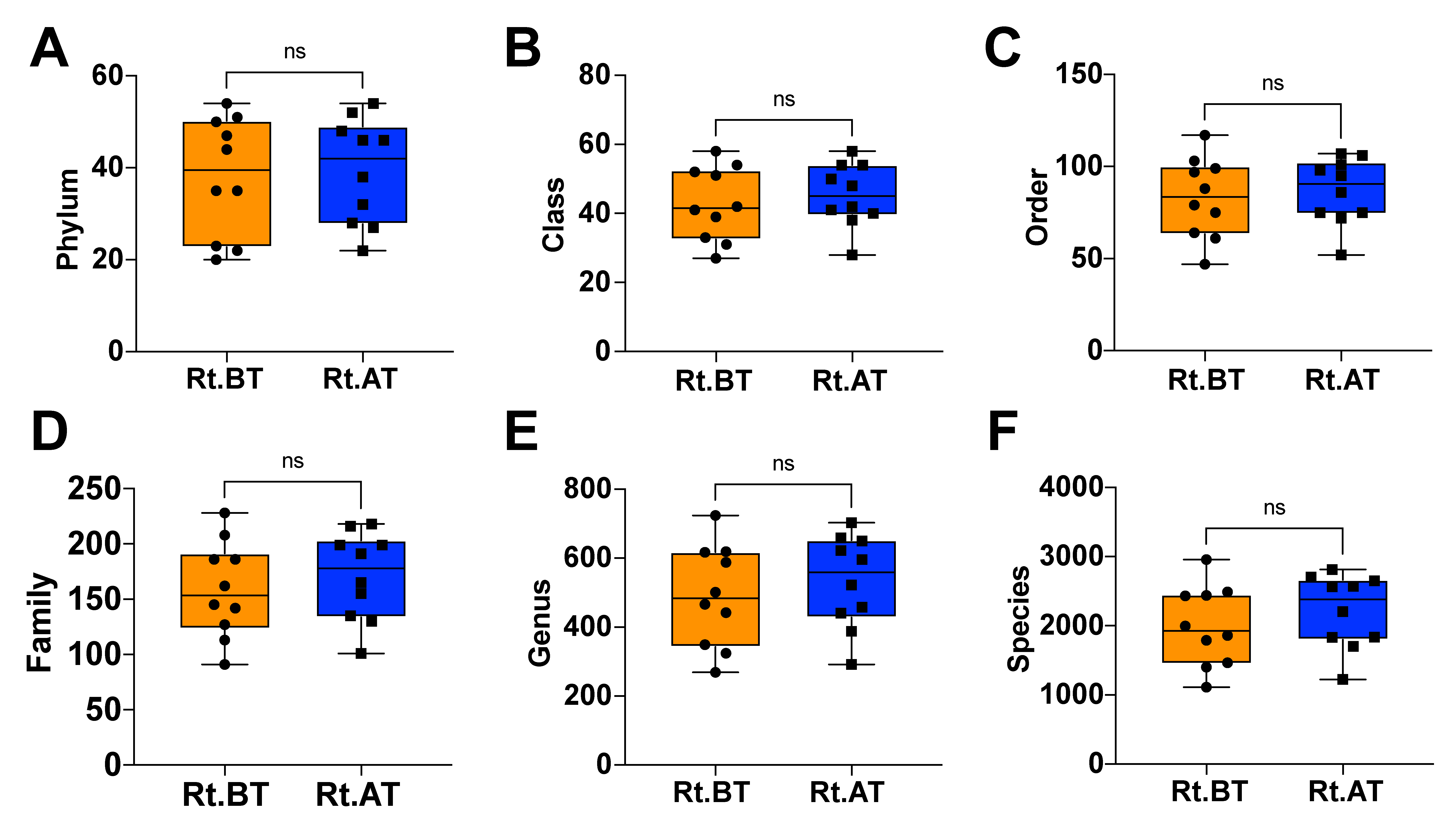


Supplementary Figure 1 Comparison of the number of species layers in patients before and after radiotherapy


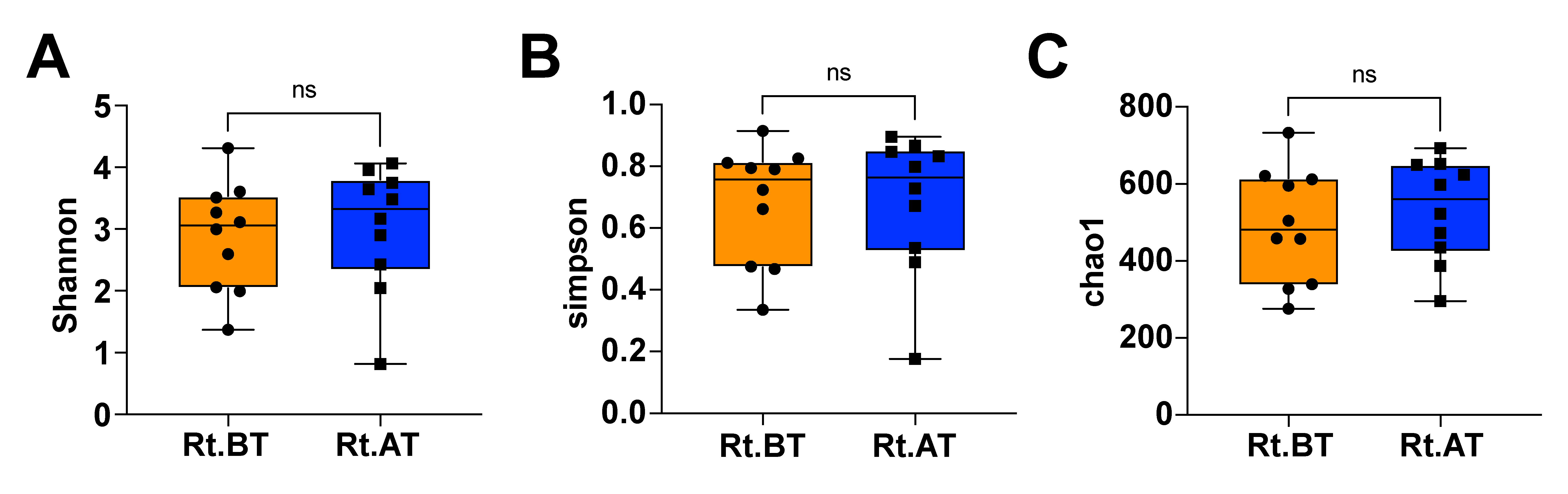


Supplementary Figure 2 Comparison of Shannon, simpson, chao1 indices of patients before and after radiotherapy

Supplementary Table 2 Species number in different levels

| Sample | Phylum | Class | Order | Family | Genus | Species |
| --- | --- | --- | --- | --- | --- | --- |
| RB.GUT1 | 35 | 42 | 79 | 145 | 442 | 1790 |
| RA.GUT1 | 38 | 48 | 95 | 191 | 596 | 2569 |
| RB.GUT2 | 20 | 31 | 61 | 127 | 349 | 1466 |
| RA.GUT2 | 27 | 40 | 75 | 155 | 458 | 1831 |
| RB.GUT3 | 47 | 41 | 88 | 162 | 501 | 1996 |
| RA.GUT3 | 46 | 42 | 86 | 165 | 522 | 2203 |
| RB.GUT4 | 35 | 39 | 75 | 142 | 466 | 1859 |
| RA.GUT4 | 32 | 41 | 75 | 135 | 441 | 1833 |
| RB.GUT5 | 50 | 51 | 97 | 186 | 617 | 2438 |
| RA.GUT5 | 54 | 54 | 101 | 199 | 650 | 2651 |
| RB.GUT6 | 51 | 54 | 103 | 208 | 619 | 2489 |
| RA.GUT6 | 48 | 50 | 98 | 199 | 622 | 2562 |
| RB.GUT7 | 44 | 52 | 99 | 186 | 588 | 2432 |
| RA.GUT7 | 46 | 54 | 106 | 216 | 659 | 2708 |
| RB.GUT8 | 54 | 58 | 117 | 228 | 724 | 2957 |
| RA.GUT8 | 52 | 58 | 107 | 218 | 703 | 2816 |
| RB.GUT9 | 22 | 27 | 47 | 91 | 269 | 1113 |
| RA.GUT9 | 22 | 28 | 52 | 101 | 292 | 1223 |
| RB.GUT10 | 23 | 33 | 64 | 113 | 324 | 1403 |
| RA.GUT10 | 28 | 38 | 72 | 130 | 388 | 1700 |
